# Native-likeness in second language lexical categorization reflects individual language history and linguistic community norms

**DOI:** 10.3389/fpsyg.2014.01203

**Published:** 2014-10-27

**Authors:** Benjamin D. Zinszer, Barbara C. Malt, Eef Ameel, Ping Li

**Affiliations:** ^1^Department of Psychology, Center for Language Science, Pennsylvania State University, University ParkPA, USA; ^2^Department of Brain and Cognitive Sciences, University of RochesterRochester, NY, USA; ^3^Department of Psychology, Lehigh University, Bethlehem, PAUSA; ^4^Faculty of Psychology and Educational Sciences, University of LeuvenLeuven, Belgium

**Keywords:** lexical categorization, lexical semantics, bilingualism, immersion, language learning

## Abstract

Second language learners face a dual challenge in vocabulary learning: First, they must learn new names for the 100s of common objects that they encounter every day. Second, after some time, they discover that these names do not generalize according to the same rules used in their first language. Lexical categories frequently differ between languages (Malt et al., 1999), and successful language learning requires that bilinguals learn not just new words but new patterns for labeling objects. In the present study, Chinese learners of English with varying language histories and resident in two different language settings (Beijing, China and State College, PA, USA) named 67 photographs of common serving dishes (e.g., cups, plates, and bowls) in both Chinese and English. Participants’ response patterns were quantified in terms of similarity to the responses of functionally monolingual native speakers of Chinese and English and showed the cross-language convergence previously observed in simultaneous bilinguals (Ameel et al., 2005). For English, bilinguals’ names for each individual stimulus were also compared to the dominant name generated by the native speakers for the object. Using two statistical models, we disentangle the effects of several highly interactive variables from bilinguals’ language histories and the naming norms of the native speaker community to predict inter-personal and inter-item variation in L2 (English) native-likeness. We find only a modest age of earliest exposure effect on L2 category native-likeness, but importantly, we find that classroom instruction in L2 negatively impacts L2 category native-likeness, even after significant immersion experience. We also identify a significant role of both L1 and L2 norms in bilinguals’ L2 picture naming responses.

## INTRODUCTION

Second language acquisition research has often highlighted the role of learners’ language history as a strong predictor of ultimate second language (L2) attainment in syntax and phonology (e.g., Flege, 1987; Johnson and Newport, 1989). Variables of interest have typically included age of acquisition (AOA) and length of residence (LOR) in a second language environment, which have good predictive value for proficiency in syntax and phonology. The roles of these predictors in lexical acquisition, however, have not been as clear when measured through the lens of vocabulary size (e.g., Snow and Hoefnagel-Höhle, 1978) or brain responses to word stimuli (see Weber-Fox and Neville, 1996; Ojima et al., 2011; Granena and Long, 2012 for several perspectives).

A closer examination of lexical semantics reveals, though, that the development of the lexicon may be more analogous to that of syntax and phonology than such divergent outcomes suggest. Recent research in lexical categorization has moved beyond the size of learners’ vocabularies and investigated more subtle aspects of word knowledge such as lexical category boundaries in both native and L2 speakers of a language. The studies reviewed below have found significant variation in lexical categorization patterns among native speakers, simultaneous bilinguals, and sequential bilinguals as a function of predictors such as age of onset, language learning experience, and usage patterns. In this paper we examine determinants of L2 lexical acquisition in more detail, with emphases on L2 immersion experience and its interaction with both individual bilinguals’ language histories and the word use patterns of the linguistic communities in which both first and second language are acquired.

### LEXICAL CATEGORIZATION

Decades of research have indicated differences in lexical categorization across languages (such as the seminal comparison of color categories by Landar et al., 1960), extending beyond abstract domains to concrete domains such as furniture, clothing, and household storage and serving vessels, and observed across Spanish, English, Chinese, Dutch, French, Russian, and more (Graham and Belnap, 1986; Malt et al., 1999, 2003; Ameel et al., 2005; Pavlenko and Malt, 2011; see Malt and Majid, 2013 for review). These differences mean that to use words as a native speaker does, language learners must acquire non-obvious, language-specific ways of generalizing names to new objects. For native speakers, fine-tuning of lexical categories may begin in infancy, but it continues beyond childhood, at least up to 14 years of age (Ameel et al., 2008), reflecting the significant challenge in language acquisition that word learning poses, even for monolinguals (see also Bowerman and Levinson, 2001). Developing adult, native-like boundaries between close competitor names requires attention to an increasing number of features of an object over time (Ameel et al., 2008). For example, no single concrete or abstract feature is sufficient to isolate members of the English category *bottle* from the set of 60 common household containers used by Malt et al. (1999). Instead, an interplay between features such as shape (typically cylindrical), material (plastic or glass), and function (containment of a fluid) define this broad category of container-like objects.

Learners of a second language, including children who acquire two languages simultaneously, are thus faced with a major incongruity between languages. For example, Chinese (referring to Mandarin Chinese throughout this paper) and English differ in the principal features by which containers are categorized. Native Chinese speakers use *píngzi* for tall, transparent beverage containers (like a 20 oz soft drink) and *guàn* for shorter, rounder, and more extended in volume, containers (like a 12 oz soft drink), analogous to the English categories *bottle* and *can* respectively. However, the relative priority of material (plastic or metal) and shape (height and roundness) as defining features differs between Chinese categories and English categories. A tall, metallic container for shaving cream may be called *píngzi* in Chinese but *can* in English, violating the ostensive translation relationships for *píngzi-bottle* and *guàn-can.*

Lexical categorization is a valuable tool for identifying variation in lexical semantic mappings among speakers, and with this more sensitive measure of lexical semantic variation, second language lexical proficiency may no longer be sufficiently described by the accumulation of a list of words as tested by most picture naming, lexical decision, and fluency tasks. Instead, lexical semantic mappings are more precisely probed when many similar objects are named, which allows inferences about the boundaries of a given speaker’s lexical category. For instance, the researcher can examine which drinking vessels are named *cup* and which similar objects receive a different name (such as *mug* or *glass*) by a speaker.

Recent work has investigated whether and how bilinguals can maintain native-like lexical semantic representations in each language despite these differences. Ameel et al. (2005) tested simultaneous Dutch–French bilinguals on the names of common containers and serving dishes. Significant influences of both Dutch and French mappings were measured in the bilinguals’ categorization patterns for both languages, and the differences between lexical categories in the bilinguals’ Dutch and French were significantly smaller than the differences between monolinguals of each language. In effect, the simultaneous bilinguals partially converged across the two languages. They achieved this convergence by shifting category centroids in each language toward one another for greater consistency between approximate translation equivalents and reducing the number of features used to define category boundaries (Ameel et al., 2009). As such, convergence produces more similar lexical categories in each language and minimizes the conflict faced by the simultaneous bilinguals in organizing the objects into named categories. Sequential bilinguals also show similar trends toward convergence (Pavlenko and Malt, 2011; Malt et al., under review). The accumulating findings in lexical categorization behavior of simultaneous and sequential bilinguals are highly suggestive of a dynamic representation for lexical semantics, mutually influenced by both languages, susceptible to change well into adulthood.

These cross-language transfer and convergence effects can be thought of in terms of how exposure to one language might change mappings from objects’ representative features to words in the other language of the bilingual speaker. Theoretical models of lexical semantic representation, such as Van Hell and De Groot’s (1998) Distributed Feature Model describe a set of underlying features whose combination may be used to define lexical concepts by linking these features to a lexical node. Models that use feature-based representations have been further adapted to accommodate broader asymmetry between languages (Dong et al., 2005) and the relative salience of different features in bilingual categorization (Ameel et al., 2009).

At least two computational models have attempted to simulate bilingual lexical categorization (Zinszer et al., 2011; Fang et al., 2013), drawing on connectionist architecture to translate language-specific mappings into training parameters for lexical nodes and high-dimensional semantic representations. These models are consistent with previous connectionist models of monolingual word learning (such as McClelland and Rogers, 2003) that rely on distributed feature representations to reproduce semantic category hierarchies (e.g., *sunfish* belongs to *fish*, which belongs to *animals*, all of which differ from *plants*) as a result of feature overlap between exemplars.

Although there are only a few quantitative accounts of bilingual lexical categorization, a number of likely predictors for development of lexical categories are apparent from the broader study of second language acquisition. The extent of L2 immersion, age of second language onset, time spent learning the second language in a formal setting (classroom training), and patterns of language use (the extent to which the languages are intermixed in use) all appear to be involved in non-native learners’ degree of success in learning a second language. Further, because name choice for an object may vary across speakers (e.g., Malt et al., 1999) the categorization norms of a linguistic community are an important means of quantifying a language learning environment, describing the variety of lexical semantic mappings used by native speakers in that community.

While many studies in second language acquisition explore the influence of language history variables on lexical learning, fewer studies have evaluated a combination of such variables simultaneously and properly controlled for interaction among the variables and statistical obstacles to measuring effects of variables individually, as outlined by Stevens (2006). None of the research to date has simultaneously related all of these variables to lexical categorization as a measure of word learning. We now consider these variables and how they may impact L2 development of lexical categorization in more detail.

### SECOND LANGUAGE IMMERSION

The value of L2 immersion is uncontroversial in second language acquisition research with respect to many components of L2 acquisition. Recent findings in lexical categorization suggest that as in other domains of language acquisition, native-like L2 lexical categorization is supported by L2 immersion. Malt and Sloman (2003) measured the English lexical categorization of 68 bilinguals (including 15 Chinese–English from various dialect backgrounds) immersed in an English environment by asking them to name pictures of common household containers, comparing the name distributions among the bilinguals to those of native English monolinguals. The bilingual participants had varying levels of English proficiency, years of English study, ages of English acquisition, and durations of English immersion. When contrasted against the other language history variables, time spent in the immersion environment was a significant predictor for the acquisition of native-like lexical semantic mappings. Immersion accounted for the greatest proportion of the variance in participants’ L2 native-likeness when entered into a multiple regression alongside years studying L2, suggesting its relative importance above formal language training. Further, age of onset and age of immersion effects were completely removed when regressed alongside length of immersion, highlighting the confounding relationships between these variables and the importance of immersion duration as a confound of age effects (Malt and Sloman, 2003).

Within-category variation arises constantly as part of the natural environment, as one may have occasion to sit in several different *chairs* each day and drink from a variety of *cups*. However, classroom learning includes little exposure to the within-category variation necessary to acquire native-like lexical semantics. Consequently, immersed learners are likely to follow different developmental trajectories than non-immersed learners, as their respective language inputs differ fundamentally in the lexical semantic domain. Additionally, aspects of language history interact or confound with immersion experience, as described in Malt and Sloman’s (2003) study above. Understanding other learning variables in concert with immersion may offer a novel perspective on L2 lexical semantic development pre- and post-immersion.

### AGE OF L2 ONSET

Age of second language onset as a predictor of eventual second language attainment remains a controversial topic, as evidence for and against a sensitive period for language acquisition is weighed alongside varying levels of other confounding age-related variables (such as years of L2 exposure, motivation, and socialization; see a recent review in Li, 2014). Age effects measured in lexical development by vocabulary size (e.g., the Peabody Picture Vocabulary Test) and translation tasks suggest that older learners may be at an advantage relative to early childhood learners (Snow and Hoefnagel-Höhle, 1978). This effect may arise in part because adults already have existing lexical semantic representations on which to base L2 word learning. One recent ERP study supports this later-is-better advantage for native-likeness of semantic processing (Ojima et al., 2011) while another ERP study (Granena and Long, 2012) indicates an advantage for earlier ages of onset lexical acquisition.

However, these tests do not account for between-language variation in lexical semantic mappings and may overlook non-native word uses by older speakers who rely on direct translation for L2 learning. The relationship between age effects and native-like lexical categorization performance is not entirely clear. Although Malt and Sloman (2003) found a weakly negative effect for later ages of L2 onset, this effect vanished after controlling for immersion. Further, other recent findings have suggested that earlier introduction of L2 may lead to reduced native-likeness of lexical semantic mappings in both L1 and L2. Very early onset Russian–English bilinguals show relatively less similarity to either L1 or L2 norms when speaking L1 compared to their later-onset peers who showed more stable influence of each language over their L1 production (Pavlenko and Malt, 2011). The very early onset bilinguals’ unique category patterns may arise from incomplete acquisition of L1 or interference of L2 in the acquisition of L1 patterns. One possible explanation is that L1–L2 interaction dramatically increases in earlier ages of onset, supported by recent computational models (Zinszer and Li, 2010; Li and Zhao, 2013; see also articles in a special issue on computational modeling, ed. Li, 2013) which have demonstrated that prior entrenchment of L1 representations may produce age effects which resemble a sensitive period and that lexical semantic representations are more integrated between languages for early onset learning, while the languages are organized relatively independently for later-onset learners (Li and Zhao, 2013).

The possible departure from conventional “earlier is better” wisdom about age of onset raises questions about whether simultaneous bilinguals are unique in their degree of convergence between languages. If late bilinguals show diminished convergence, more native-like representations may be learnable in both L1 and L2 independently, even when marginal cross-language transfer is observable.

### L2 CLASSROOM INSTRUCTION

Malt and Sloman’s (2003) study of L2 English learners found that formal training in English prior to immersion offered no predictive power after accounting for years of L2 immersion. Based on this result, L2 training would seem to have minimal value for acquiring native-like L2 lexical semantic mappings. However, some degree of successful L2 lexical semantic remapping has been observed in non-immersed learners with sufficiently advanced L2 education. Chinese students in their third year of undergraduate study as English majors demonstrated significantly higher L2 native-likeness in semantic similarity judgments than a first-year cohort (Dong et al., 2005).

The latter result does not strongly contradict the Malt and Sloman (2003) finding, however, in that the Chinese students of English at both levels still relied primarily on their native Chinese semantics when making English judgments, showing greater similarity to the monolingual Chinese speakers than to English–Chinese bilinguals (native English speakers). Both the improvement toward slightly more native-like English associations and the general bias toward Chinese semantics are reflected in the learners’ significant convergence, producing semantic similarity judgments that were more similar across languages than the judgments between the Chinese monolinguals and English–Chinese bilinguals. For these sequential bilingual learners, language systems interacted to allow a small degree of transfer of learned L2 mappings onto L1 while never overcoming the overall L1-likeness of the representations in both languages. Thus the role of classroom experience in acquiring native-like L2 lexical categorization deserves more scrutiny.

### LANGUAGE USE CONTEXT

The type of language experience gained in an immersion environment can vary substantially among bilinguals. Simultaneous bilinguals, such as those in Ameel et al.’s (2005) study are often immersed in an environment that involves frequent input from speakers of both languages. Sequential bilinguals may transition from a monolingual L1 environment to a new language environment where most speakers are monolinguals of L2. In this new environment, L1 use may be limited to a social or familial community, and L2 use may be primarily for work or business.

The monolingual or bilingual context of the language environment or the extent to which speakers switch between languages changes the degree of cognitive control necessary for language production. Specifically, highly bilingual environments raise the potential for frequent code-switching and increase activation of the non-target language, which must then be actively inhibited from production (Green and Abutalebi, 2013). This effect arises from the persistent simultaneous activation of languages (see Kroll et al., 2006, 2012 for reviews) and creates the possibility that each language may be susceptible to change through retrieval induced reconsolidation (Wolff and Ventura, 2009). In retrieval induced reconsolidation (see Forcato et al., 2007), all active representations are adjusted during access by the current input, even if not selected. Because lexical semantic mappings draw on shared cross-language conceptual representation (Van Hell and De Groot, 1998), production of one language may result in reshaping of the other, particularly when both languages are highly active in bilingual environments with more frequent code-switching.

Evidence supporting the view of language change through use can be found in a recent study of phonological accent in the native language. De Leeuw et al. (2010) identified code-switching as a significant predictor in the extent to which the first language phonological system was preserved for bilinguals immersed in an L2 environment. Specifically, greater time spent in L1 environments that inhibited code-switching (such as written correspondence and professional settings) was a significant predictor of L1 stability, while time spent in L1 environments that were permissive of code-switching (e.g., among family and friends) was not associated with preservation of L1 phonology. De Leeuw et al.’s (2010) finding is highly suggestive of the role of language use in regulating the contact and transfer between L1 and L2. Such findings may be relevant to the observation of substantial convergence in simultaneous bilinguals’ lexical categorization behavior found by Ameel et al. (2005). Although Ameel et al. (2005) did not directly measure the incidence of intra-sentential code-switching in this setting, the highly bilingual environment is one in which code-switching is more likely to occur.

The contrast observed in Pavlenko and Malt’s (2011) early and childhood bilinguals may also reflect the influence of contexts of language use. The early bilinguals in their study (age of L2 onset 6 years or earlier) reportedly participated in a much more fluid bilingual environment from the outset than the child bilinguals (age of L2 onset 8–15 years). These patterns of use are confounded with the age of onset and incomplete L1 acquisition effects and may explain the differences between these groups in native-likeness of L1 and L2. Later-onset of L2 correlates with more discrete separation between language environments and therefore relatively more native-likeness, even as cross-language influence begins to appear.

### LINGUISTIC COMMUNITY NORMS

Because native, monolingual speakers of a language also show significant variation in lexical categorization patterns, even monolingual infants acquiring their native language are exposed to variable input for many objects’ names. In the relatively familiar domain of household containers, Malt and Sloman (2003) found a broad range of native speaker agreement levels across objects, with the dominant name being produced by as few as 43% of native speakers for some objects (the remaining 57% divided between two or more subordinate names) and 100% agreement for others. In effect, immersed learners are exposed to an array of potential names for many objects, and for some objects the most dominant or native-like name arises in only a minority of encounters (native agreement levels below 50%). L2 learners are thus challenged with determining to *which* of several new categories an object is best suited. This ambiguity results in a many-to-many mapping problem for a single object. For example, a particular serving vessel may be called *diézi* by 70% of Chinese speakers and *pánzi* by 30% of Chinese speakers. Both names may be translated as *dish* or *plate* in English, and *diézi* has the further possible translation of *saucer*. In effect, the Chinese–English bilingual may encounter at least five unique categories of varying fitness for this object from native speakers of the two languages.

As we have discussed earlier, bilinguals’ lexical categorization patterns in either language are, indeed, jointly predicted by the native (monolingual) patterns of the two languages (Ameel et al., 2005, 2009; Pavlenko and Malt, 2011; Malt et al., under review). At the earliest stages of learning, before they develop sufficiently elaborated L2 representations, L2 learners draw heavily on L1 representations for production (see the Unified Competition Model of MacWhinney, 2012). These early learners’ L2 categorization patterns should reflect their confidence in L1 naming (i.e., the extent of L1 dominant name agreement) because, in the absence of L2-specific lexical semantic knowledge, inferences about L2 words are based on knowledge of their L1 translation equivalents. Eventually L2 learners become sequential bilinguals, cross-language influence approaches that of simultaneous bilinguals, and they become less native-like in their L1 as L2 lexical semantic proficiency increases under immersive L2 influence (Pavlenko and Malt, 2011; Malt et al., under review). Typicality ratings also can be construed as a measure of native speakers’ confidence about the name of an object, and Pavlenko and Malt (2011) found that Russian–English bilinguals relied on both Russian and English native typicality norms for individual objects when naming these objects in Russian, suggesting that their intuitions about categorization were influenced by the perceived confidence of each language community in an object’s category membership.

It is evident that in many instances of simultaneous and sequential bilingualism, the category information provided to bilinguals by the native-speaker communities of each language is variable and yet still bears a significant influence on their production in both languages. With relatively few lexical category stimulus sets normed for native speakers of more than one language and tested on sufficiently advanced bilinguals of both languages, the exact degree and means of this cross-language influence remains to be explored. However, native category norms that represent the full distribution of names produced and thus the degree of name agreement and variation among native speakers may allow an elaborated view of cross-language competition and transfer. The extent to which L1 representations are vulnerable to change may vary as a function of their own entrenchment, with greater native naming agreement representing more robust L1 representations. Conversely, objects named with greater consistency in L2 (high L2 native agreement) could be associated with better learning outcomes as compared to objects for which L2 speakers show little agreement.

### THE PRESENT STUDY

In the present study, we aim to disentangle the respective roles of four broad categories of individual language history variables in predicting native-likeness of L2 lexical semantics: L2 environment (non-immersion vs. immersion), age of L2 onset, years of L2 classroom study, and L2 usage pattern [code-switching frequency (CSFreq)]. Collinearity between age and immersion predictors has been shown to cause serious confounds in studies of second language acquisition (see Stevens, 2006 for detailed analysis). Recent ERP studies of individual L2 word processing have identified both positive (Ojima et al., 2011) and negative (Granena and Long, 2012) effects of age while trying to deconfound the effects of age, exposure, and immersion. Previous studies of lexical categorization have also identified confounded relationships between LOR in an L2 environment and age of onset (Malt and Sloman, 2003) and between age of immersion and patterns of language use or dominance (Pavlenko and Malt, 2011).

By measuring several language history variables together, accounting for the earliest L2 exposure (that is, L2 onset before immersion), and using categorization as a more sensitive measure to inter-personal lexical semantic variation, we aim to make better statistical estimates of each variable’s effect. We offer a simultaneous measure of four variables based primarily on the self-reports of Chinese–English bilinguals resident in Beijing, China and in Pennsylvania, United States.

We also introduce linguistic community norms for word use in L1 and L2, derived from native speakers of each language, as possible predictors of bilinguals’ lexical categorization patterns. The contribution of such norms has rarely been considered in predicting L2 performance (except see Pavlenko and Malt, 2011).

These non-immersed and immersed participants are compared in an L2 (English) lexical categorization task that has proved highly sensitive to variation in lexical semantic mapping for other populations of bilinguals. Based on the simultaneous evaluation of all four language history variables and the linguistic community norms, we evaluate participants’ English native-likeness on the lexical categorization task. We offer an interactive account of how various aspects of one’s native language, second language, and language learning history jointly influence the lexical semantic mappings that defines object naming, a behavior that occurs often in our daily experience.

## MATERIALS AND METHODS

### PARTICIPANTS

Two groups of bilingual students, one in the United States and one in China, participated in this study. In the U.S., Chinese–English bilingual undergraduate and graduate students were recruited from the Introduction to Psychology subject pool and through posters around the campus community at Penn State University (State College, PA, USA). In China, Chinese–English bilingual undergraduate and graduate students were recruited through an online campus message board (BBS) and through personal referrals at Beijing Normal University (Beijing, China). Generally speaking, the students at Penn State were slightly younger (mostly undergraduates) than those at Beijing Normal (mostly graduate students), were first exposed to English at a slightly earlier age, and had higher self-rated proficiencies in English.

Although many of the bilingual participants reported some degree of training in a third language, most rated themselves at very low proficiency. Participants who self-reported a proficiency of 2.5 or greater in the third language on a 7-point scale (averaged across four ratings: reading, writing, speaking, listening) or failed to provide a proficiency rating in their third language were not included in the data. In total, 57 participants from Beijing Normal and 68 participants from Penn State met the inclusion criterion. Third languages included French, German, Russian, Mongolian, Japanese, Korean, Taiwanese, and Cantonese.

Penn State students ranged in age from 18 to 23 (*M*= 19.5, SD = 1.2). They were first exposed to English between ages 1 and 16 (*M*= 8.2, SD = 3.7), and self-rated their English proficiency between 2.5 and 7.0 (*M*= 4.7, SD = 1.3). The Penn State students had resided in the United States for 0–19 years (*M*= 5.0, SD = 5.9). Students at Beijing Normal University had ages ranging 18 to 28 (*M*= 22.8, SD = 2.0), and age of earliest English exposure was 5–15 (*M*= 11.4, SD = 2.1). Their self-rated English proficiency varied between 1.3 and 5.5 (*M*= 3.9, SD = 1.0), as some were studying English while others majored in different subjects. None of the participants at Beijing Normal University reported living in or visiting an English-speaking country for an extended period of time.

We also drew on a set of native-speaker norming data from functionally monolingual participants who had participated in a previous version of the lexical categorization task, using the same stimuli (Malt et al., 2013). The picture naming data for 25 native Chinese speakers in China and 28 English speakers in Pennsylvania provided linguistic community norms for the current analyses. Their choices represent the most likely input patterns for bilinguals in their respective language environments.

### MATERIALS

All participants completed a language history questionnaire (LHQ; Li et al., 2006) to assess bilingual status, L2 proficiency, age of L2 acquisition, and behavioral predictors such as patterns of code-switching. The LHQ was available in both English and Chinese (simplified characters) and administered according to the dominant language environment. With respect to code-switching, the LHQ allows participants to self-rate their frequency of code-switching in four contexts: Spouse & Family, Friends, Co-Workers, and Classmates. Participants ranked their CSFreq in each context ordinally, using response options that ranged from “Rarely” to “Very Frequently.” These responses were transformed into a Likert score between 1 and 5 and averaged within context group to produce the CS scores.

Early trials at Penn State revealed that several participants failed to complete the code-switching section of the LHQ or claimed to never code-switch, a self-report that may (in some cases) underestimate the true rate of code-switching in cultural environments that stigmatize language mixing. An additional code-switching questionnaire (CSQ) was added to subsequent sessions to specifically probe participants’ code-switching and was administered according to the dominant language environment. A single item on the CSQ was used to obtain a point-estimate of participants’ overall CSFreq: “Do you use English words when speaking Chinese, or do you use Chinese words when speaking English?” rated on a five-point ordinal scale with response options from “never” to “very often.”

**FIGURE 1:**
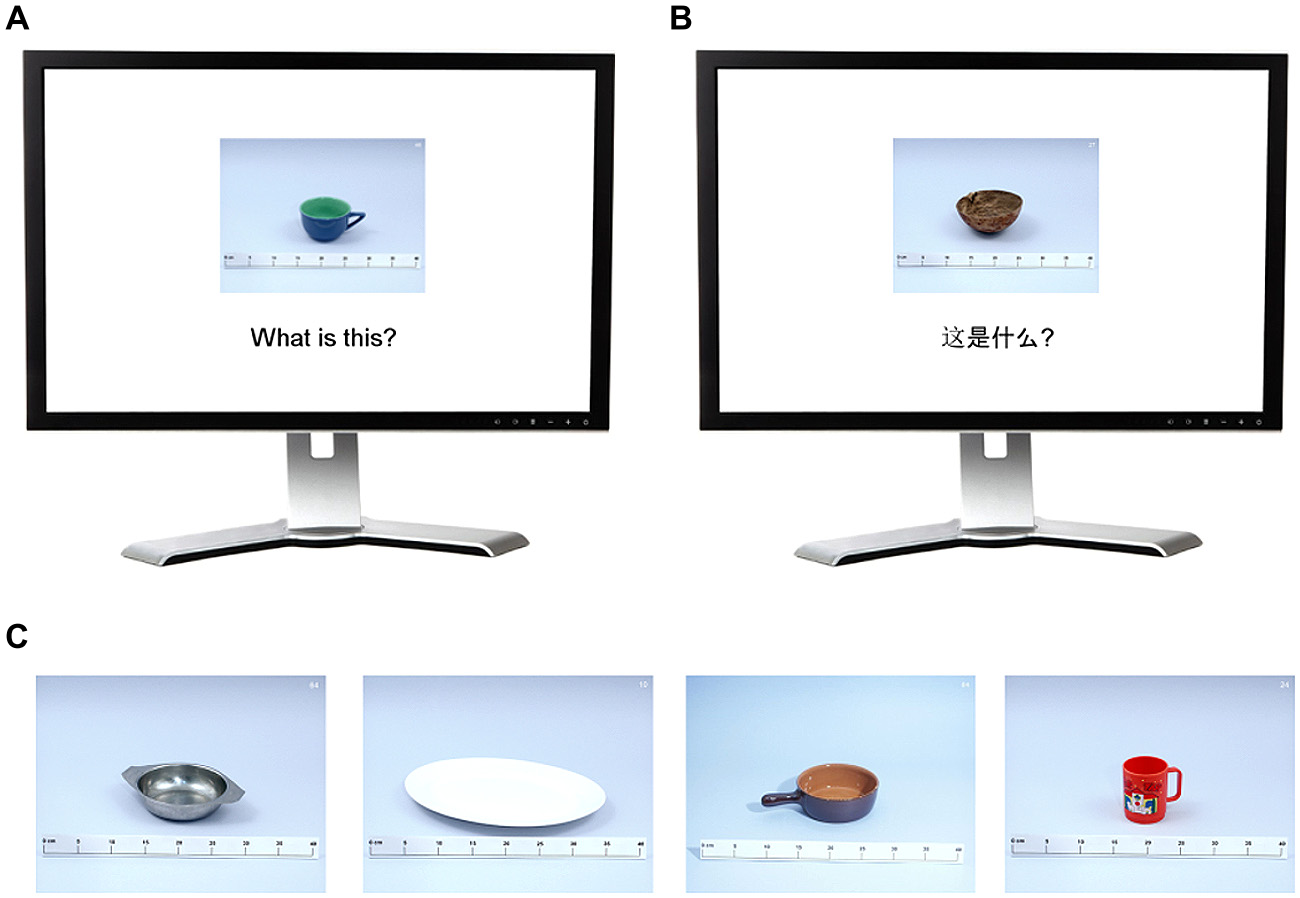
**Sample displays for English (A) and Chinese (B) naming tasks depicting two example objects from the dishes stimulus set. (C)** Additional example objects. Participants viewed all 67 objects in this format with the text prompt (according to task language) provided below each item.

Sixty-seven photographs of common household objects were used to elicit category names from monolingual and bilingual participants. These objects were drawn from a stimulus set (called the dish set) used by Ameel et al. (2005) to reveal cross-language lexical categorization differences in Dutch–French bilinguals. Each photograph contained a single household serving vessel (e.g., a plate, cup, or bowl) on a neutral background and a centimeter ruler in the foreground for scale (see **Figure 1**). Photographs were displayed at 480 × 360 pixels on a personal computer equipped for digital recording. Each voice response was recorded through a standard omni-directional consumer microphone to the computer’s sound card and encoded as 10 s uncompressed WAV files. Each photograph was accompanied by the written prompt: “What is this?” or 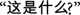 according to the task language.

An Operation-Span (O-Span) test was also used to screen the bilingual participant groups for systematic differences in working memory, a cognitive factor that might be confounded with language proficiency or language transfer. The O-Span includes mathematical and verbal components (Turner and Engle, 1989): Participants judge the accuracy of math equations and are provided a word to remember after each judgment. After several math and word combinations, participants are prompted to recall the words they have seen. Arabic numerals were used for the math component (consistent with both Chinese and American math education) and Chinese characters were used for the verbal component. Participants entered their judgments using a computer keyboard and recorded their verbal responses on a paper worksheet. No significant difference was found between the two bilingual samples in their O-Span scores.

### PROCEDURE

After giving informed consent in the local language, participants completed the LHQ, also in the local language (Chinese or English). They then completed an unrelated English receptive vocabulary task (results not presented here) to establish an English language mode to the extent possible in both the Chinese- and English-immersed participants. After the vocabulary test, all participants performed the English picture naming task. The Chinese O-Span was then completed and used to shift participants into a Chinese language mode before naming the objects again in Chinese. Finally, the CSQ was completed last. Participants in the US completed English and Chinese tasks on separate days, 1–2 weeks apart (range: 6–21 days; mean: 9 days) and counter-balanced for order. Sessions in China could not be scheduled separately and all tasks were completed on the same day, with English first, followed by Chinese. We reasoned that the English task was less likely to influence Chinese naming in a Chinese immersion environment, and intervening Chinese tasks (namely, the O-Span) would help to reduce any language priming effects.

In the picture naming tasks, participants were instructed to name aloud photographs of objects depicted on the computer. They were asked not to name the objects’ contents, as illustrated by two photographic examples: a grocery bag full of vegetables (called *bag*) and a trash can full of paper (called *trash can*). These instructions were provided in written form on the computer screen according to the language of the task. Participants were verbally encouraged to name every object and to always make a guess if unsure. Participants were also provided two practice naming trials for photographs of unrelated bottle-like stimuli, followed by the most dominant monolingual name for each stimulus ( or 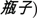 to demonstrate the desired response type. Participants were permitted to take as long as needed to name each picture to ensure that they selected what they considered the best name for each object. Due to disk storage constraints, only the first 10 s (from the onset of the stimulus) of participants’ responses were digitally recorded by the computer for each stimulus.

### DATA ANALYSIS

Participant responses were transcribed from audio recordings by high-proficiency Chinese–English bilinguals in the United States who were able to comprehend Chinese responses and phonetically accented English responses. Transcribers were not able to view the objects during transcription to prevent bias on ambiguous recordings. Transcribed responses were subsequently reduced to head nouns (e.g., “a small blue bowl” is reduced to “bowl”) for comparison with the native norming data. Skipped trials, inaudible responses, and irrelevant responses (e.g., “I don’t know”) were entered as blanks and treated as missing data.

Four biographical variables were included for each subject: Age of first exposure to English (AOEE), LOR in the English immersion environment (LOR), self-reported frequency of code-switching between Chinese and English (CSFreq) and the total number of years spent learning English (current age minus the age of first exposure, YrsLearn). For participants who failed to complete some language history and code-switching questions, missing data for the CSFreq variable were replaced with the sample mean (3% of the participants included in the analysis). Participants who did not report AOEE were excluded from the analysis (eight participants), and an additional set of early childhood bilinguals (AOEE < 5 years, six participants) were removed from the US participant data to maintain comparability with the sample in China (AOEE range 5–15 years).

Given the above exclusion/inclusion criteria for data analysis, our data analyses presented in the Results section were based on a total of 30 participants from China and 33 from State College (see **Table 1**). Results from 20 participants in the US sample and 20 in the China sample were discarded due to recording equipment failure (no audio data recorded). Participants were encouraged to speak loudly and clearly directly into a desktop microphone; however, additional participants (not excluded due to recording failure) periodically produced inaudible responses or no response, decreasing their total response rate^1^. Some participants may have chosen not to respond to stimuli when uncertain about those objects’ names, a possibility we tested by correlating response rate to self-rated English proficiency. Indeed, response rate in English was weakly correlated to English proficiency (*r* = 0.24, *p* = 0.047) while response rate in Chinese was not (*r* = 0.20, *p* = 0.101). Non-response as a predictor of name uncertainty is preserved in the remaining participants (50% or higher response rate) insofar as all trials are included for analysis, with non-response trials counted as incorrect names.

**Table 1:** Demographics and language histories of participants before and after screening.

Sample	*n*	Age (SD)	AOEE (SD)	EngProf (SD)	CSFreq (SD)	LOR (SD)
**All participants**						
Beijing Normal	57	22.9 (1.8)	11.6 (1.9)	4.1 (1.0)	1.1 (1.2)	0
Penn State	68	20.9 (2.9)	8.8 (3.3)	4.7 (1.1)	1.8 (1.2)	3.8 (5.2)
**Included participants**						
Beijing Normal	30	22.8 (1.7)	11.5 (2.2)	4.2 (1.1)	0.95 (1.1)	0
Penn State	33	21.8 (3.3)	9.8 (2.6)	4.7 (1.0)	1.9 (0.8)	2.2 (2.6)

## RESULTS

In the following sections, we present a set of analyses that examine the lexical categorization patterns of the Chinese–English bilingual participants at three different levels, as follows. (1) The group-wise analysis compares the overall patterns of transfer and convergence between Chinese and English as spoken by the bilingual participants. This analysis looks at the overall trends in naming distributions generated by sub-groups, which is defined by their degrees of L2 immersion (see details below). This analysis allows direct correlations of the bilinguals’ overall patterns with the monolingual norms. (2) The subject-wise analysis focuses on individual bilingual participants’ language histories and how these variables predict their individual differences in L2 naming patterns. (3) The item-wise analysis examines naming performance on each object of the stimulus set, controlling for variation in individuals’ language histories and examining the impact of linguistic community norms on the bilinguals’ accuracy in producing the native preferred L2 names.

### GROUP-WISE ANALYSIS: CROSS-LANGUAGE TRANSFER AND CONVERGENCE

For group-wise comparison, participants were organized by three discrete values of LOR to describe three types of immersion conditions observed in our sample: No Immersion, Short-term, and Long-term. No Immersion was defined by LOR = 0, describing participants who have never lived in an English immersion environment. English-immersed participants were divided into two groups by a median split (median non-zero LOR = 1.3 years). Short- and Long-term Immersion were defined as the samples below and above the median, respectively.

**FIGURE 2:**
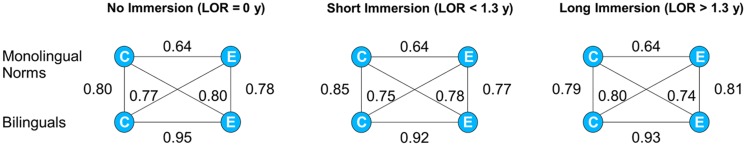
**Cross-language correlation matrices for each Immersion group, representing the correlation between lexical categorization patterns in Chinese (C) and English (E) for the bilinguals and native speaker norms**.

A cross-language correlation matrix was calculated for each bilingual and monolingual group according to the method of Malt et al. (1999; see also Ameel et al., 2005), in which the naming distribution for each object over all possible names is correlated with the naming distribution for every other object. This method produces a 67 × 67 correlation matrix with 2211 unique values (per speaker group) for our data, indicating, for each possible pair of objects, to what extent the same names were produced with the same frequency by the speaker group. These inter-object matrices can then be correlated between languages or groups, representing the degree to which objects names are distributed similarly in the two samples (regardless of the actual names themselves). **Figure 2** provides these correlation matrices for each immersion group, compared with the monolingual speakers of Chinese and English and between the Chinese and English patterns produced by the bilinguals, according to the convention of Ameel et al. (2005).

The cross-language correlations revealed that native, monolingual speakers of Chinese and English correlate in their categorization of this set of objects at *r* = 0.64 (see the top row of **Figure 2**). This value serves as the baseline correlation against which bilinguals’ Chinese and English categorization patterns can be compared. All of the bilinguals showed a highly convergent pattern of naming between languages, correlating their Chinese and English word use around 0.92–0.95 (the bottom row of **Figure 2**), strongly suggesting that they relied on a single set of mappings (with varying degrees of influence from each language). Correlation of the bilinguals’ English naming with the monolingual norms (the right-most vertical connection for each matrix in **Figure 2**) was compared using Cohen and Cohen’s (1983) method for comparing correlation coefficients. Similarity to the English norms was highest in the Long Immersion group (0.81, compared to 0.78 and 0.77 in the No and Short Immersion groups respectively, *p* < 0.01 in both cases). The bilinguals’ English categorization also decreased in its dependence on Chinese norms with increased immersion (No Immersion: 0.80 vs. Short Immersion: 0.78, *p* = 0.038; No Immersion vs. Long Immersion: 0.74, *p* < 0.001).

Surprisingly, the No Immersion group showed the highest convergence between their two languages (0.95), a relatively low correlation with the monolingual Chinese (0.80) compared to their recently immersed peers (Short Immersion, 0.85, *p* < 0.001) and greater Chinese resemblance to the English patterns (0.77, compared to Short Immersion 0.75, *p* = 0.058). This effect may be attributable to differences in the administration of the Chinese naming task, in which the No Immersion group completed Chinese naming shortly after the English naming task. Henceforth, we will examine English naming only, as English names were not subject to priming across tasks because English naming occurred either first or in a separate session for all participants, and L2 acquisition is the focus of the current study.

### SUBJECT-WISE ANALYSIS: THE ROLE OF LANGUAGE HISTORY VARIABLES

Participants’ picture naming responses were compared to a set of English native norms to generate a score for each participant describing the English native-likeness of their lexical categories. Each of a participant’s responses was awarded a score based on the proportion of native monolingual speakers who produced that same response in the norms (following Malt and Sloman, 2003). Thus if an object was called *mug* by 75% of the norming group and *cup* by 25%, the bilingual participant would receive 0.75 points for naming the object *mug*, 0.25 for *cup*, and 0 for anything else. These point values were averaged across the 67 objects for each participant, rendering an agreement-weighted native-likeness score ranging between 0 and 0.68 (the mean of agreement level for native English speakers across all objects).

We estimated a linear regression model for the English native-likeness scores over participants’ language histories to determine the relationships between language background and attained L2 lexical category proficiency. Previous analyses from smaller datasets showed several two-way interactions between the language history variables and an inter-dependency of the significance of these interactions in the model (see Zinszer et al., 2012, 2013 for examples). Consequently, the initial model was estimated with all possible interactions (up to four-way) and, indeed, yielded several highly significant three-way interactions [omnibus test: *F*(15,46) = 2.14, *p* = 0.02, Adjusted *R*^2^ = 0.22].

In an attempt to improve the parsimony of this model without discarding important interaction effects, an automatic Akiake information criterion (*AIC*) stepwise procedure was adopted which started with all possible interactions and systematically excluded and re-included variables to find the best-fitting model with the lowest *AIC* score (Venables and Ripley, 2002). This method produces a reduced model while minimizing impact on the model’s fitness to the data. Finally, the AIC search excluded only the four-way interaction term, resulting in a significant reduced model [omnibus test: *F*(14,47) = 2.27, *p* = 0.02, Adjusted *R*^2^ = 0.23] with slightly improved parsimony (initial model: 16 terms, *AIC* = -103.9; reduced model: 15 terms, *AIC*= -105.0). Ultimately, all predictors were included in one or more significant interactions.

**FIGURE 3:**
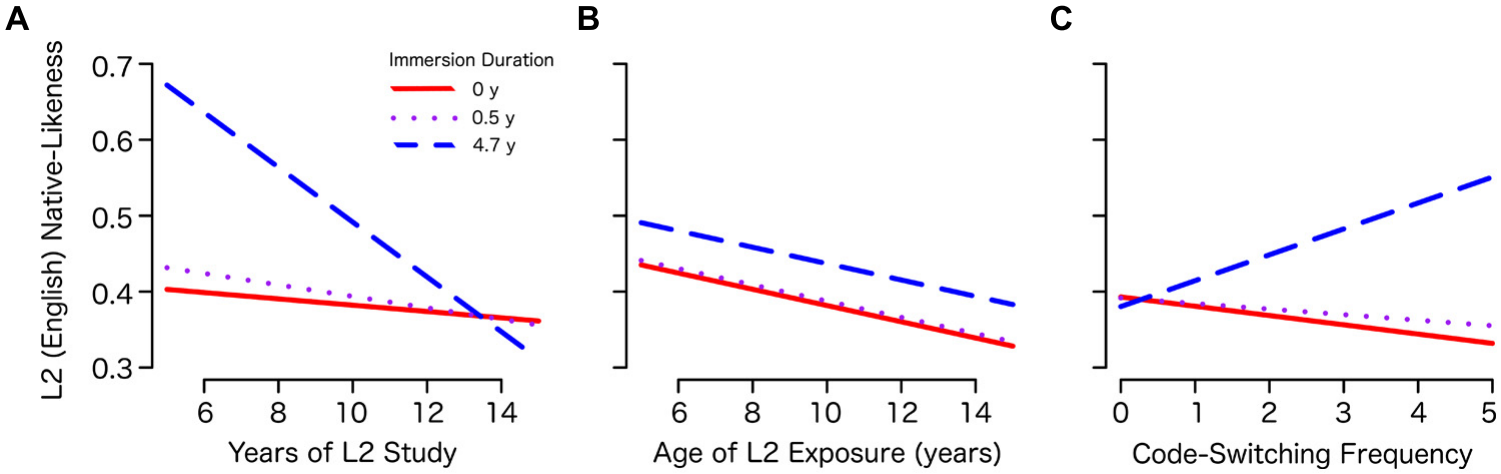
**Two-way interaction plots for Immersion and each of the learner variables. (A)** Years of L2 study. **(B)** Age of earliest L2 exposure. **(C)** Self-reported frequency of code-switching.

To understand the highly interactive terms of the subject-wise model, we generated several estimated marginal means plots based on the model’s predicted English native-likeness scores across a range of values for the two-way interactions between L2 immersion (LOR) and each remaining predictor (while holding other predictors constant at the mean value). These two-way interactions were all highly significant (LOR × YrsLearn: *p* = 0.002; LOR × AOEE: *p* = 0.014; LOR × CSFreq: *p* = 0.007). To further simplify the plots, we again used the three discrete values of LOR to describe three types of immersion conditions observed in our sample: No Immersion (LOR = 0), Short- (LOR < 1.3 years), and Long-term (LOR > 1.3 years). Short and Long-term Immersion were represented by the mean LOR values for each of these two groups: 0.5 and 4.7 years, respectively. **Figure 3** shows plots for the interactions between LOR and each of the remaining predictors: AOEE, CSFreq, and YrsLearn.

The first plot (**Figure 3A**) contrasts years of English study with the duration of English immersion, which are not independent predictors. That is, as the duration of immersion (LOR) increases, so do the years of English study (YrsLearn). Conversely, however, YrsLearn may increase without immersion experience. Therefore these variables contrast the predicted English native-likeness associated with varying durations of study when the amount of that time spent in an Immersion environment is held constant (in this case at 0, 0.5, or 4.7 years). In all three LOR conditions, the relationship between YrsLearn and English native-likeness is negatively sloped, indicating a relative disadvantage for years of English study after controlling for years of English immersion. In other words, every additional year of English study in China beyond a participant’s immersion experience reduced the native-likeness of their English categorization patterns. For example, a learner with 15 years of study and almost 5 years of immersion (LOR = 4.7) has had over 10 years of English study in China, and they are predicted to perform worse (on average) than somebody with fewer years of English study and the same amount of (or even less) immersion.

Age of earliest English exposure (AOEE; **Figure 3B**) also displayed a negative relationship with English native-likeness. When controlling for the other variables, later ages of English onset generally result in poorer performance in English categorization. Interestingly, however, the interaction with LOR did not appear to be large (the lines are roughly parallel) indicating a largely additive effect of these two variables. The relative weight of each variable was approximately balanced such that the negative effect of being exposed to English 1 year later is offset by the benefit of 1 year of immersion experience.

Participants’ self-reported CSFreq was also a significant predictor in the model and significantly interacted with immersion. As **Figure 3C** indicates, the effects of CSFreq were relatively small for non-immersed learners and learners with relatively little immersion experience, and greater CSFreq was associated with less L2 achievement. However, CSFreq was a much stronger predictor for learners with longer immersion experiences, and the direction of the influence was opposite, showing significant gains in English native-likeness with greater frequency of switching between languages. This interaction may suggest that CSFreq is most predictive for people who are immersed in the L2 environment.

### ITEM-WISE ANALYSIS: THE IMPACT OF LINGUISTIC COMMUNITY NORMS

In this analysis looking at how native naming consensus for objects impacts the likelihood of naming objects correctly, we compared each response by the participants to the single dominant name^2^ produced by the English native norm for each given object. Thus, trials in which the participant produced the norm’s dominant name were scored as 1 (correct), while all other trials were scored as 0 (incorrect). Next, we performed two binomial logistic regressions to estimate the probability that a participant would produce the dominant name for any given object.

In the first logistic regression, we entered the same language history variables used in the subject-wise analysis to determine how adequate these variables were for identifying variation in native-like categorization for different objects. The logistic regression model including only participants’ language history information contained several statistically significant predictors, but offered a very poor fit to the data (Nagelkerke *R*^2^ = 0.02, indicating that subjects’ language backgrounds could account for overall trends in the native-likeness of their English categorization but not for most of the variation trial-to-trial. This result points to the importance of considering variation in the learner’s input across objects (such as the native norms) as a predictor of success in naming individual objects.

In the next analysis, we added four language variables which described the native speaker norms for every given object: naming agreement in Chinese (L1), naming agreement in English (L2), number of alternative names produced by the Chinese norming group, and number of alternative names produced by the English norming group. Due to computational limitations, this model was estimated with up to four-way interactions and reduced using the same *AIC* stepwise search procedure described in the subject-wise analysis. The resulting reduced model improved the *AIC* compared to the initial model and included 36% fewer terms than the initial model without a serious decrease in fitness (initial model: 163 terms, *AIC*= 5036.52, Nagelkerke *R*^2^ = 0.25; reduced model: 104 terms, *AIC*= 4951.7, Nagelkerke *R*^2^ = 0.24).

As in the subject-wise analysis, the model contained many interaction terms that impeded interpretation without isolating a few of the variables. Again, we sought to describe how immersion experience affected the role of these language variables in predicting the participants’ success in producing native-like English names for objects. A binomial logistic regression predicts the probability that an outcome will occur, in this case the probability that the participant will produce the English native-like dominant name for a given object. Again, we estimated plots in which the individual variables (this time, language variables) interacted with three levels of immersion while holding all other variables constant at a mean value.

**FIGURE 4:**
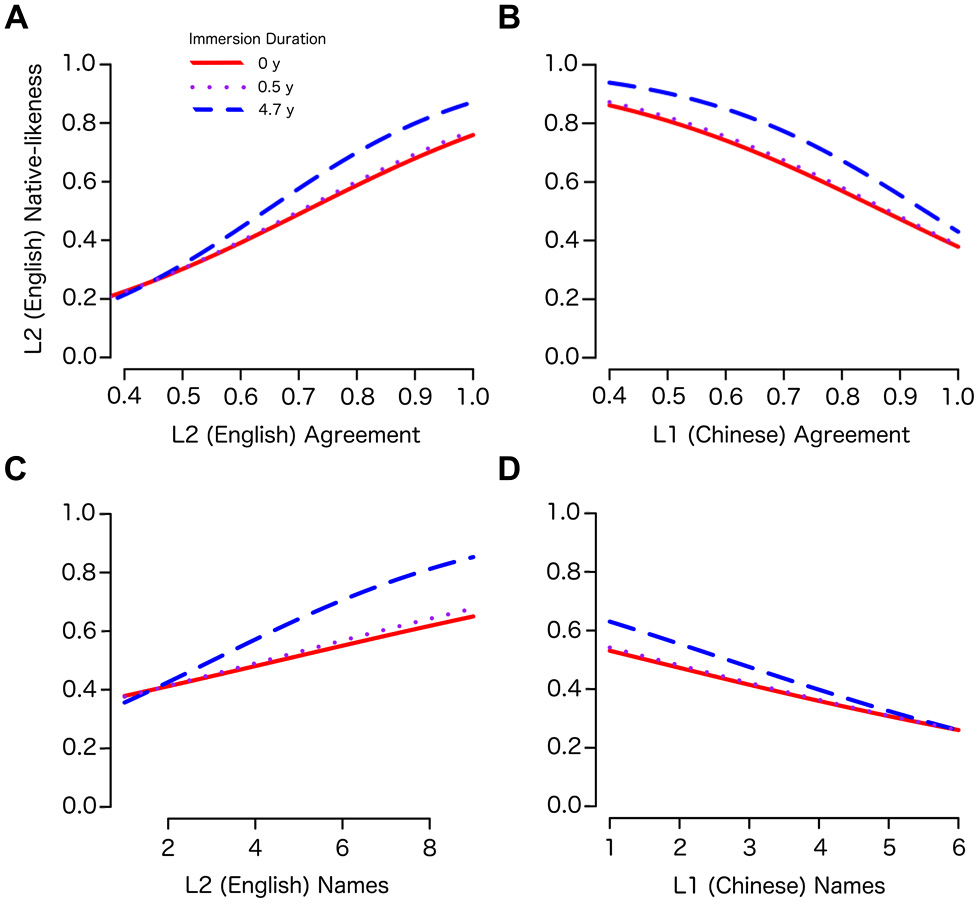
**Two-way interaction plots for Immersion and each of the linguistic community norm variables. (A)** L2 native speaker agreement (percent of a norming sample who produced the dominant name) **(B)** L1 native speaker agreement. **(C)** Number of alternate names for an object produced by the L2 native speakers in a norming sample. **(D)** Number of alternate names produced by L1 norming sample.

**Figure 4** presents plots of each linguistic community variable against the three levels of English immersion (None, Short-term, and Long-term). These plots revealed that English native-likeness in the learners was more likely at higher levels of English norm agreement (**Figure 4A**), while the inverse was true for Chinese: There was less English native-likeness with higher agreement in the Chinese norm (4B). An opposing relationship was also observed for the number of alternative names available from the norming sample. Having a greater number of English names available in the norm actually increased the predicted probability that the learners would produce the dominant English name (4C), but having many possible Chinese names for an object decreased the predicted probability of the participants producing an English native-like name (4D). The apparent advantage for a greater number of English names is explored in the next section.

In a follow-up analysis, we asked how L1 and L2 norms might interact with one another in predicting a learner’s success in producing the L2 dominant name. Several interaction terms between these norming variables were highly significant, so we examined the cross-language relationships between L1 and L2 agreement and number of alternate L1 and L2 names. This analysis offers a closer examination of two interesting effects from the preceding results: (1) native speaker agreement in each language appears to compete in predicting L2 native-likeness and (2) an increasing number of names in English seems to be associated with greater L2 native-likeness.

**FIGURE 5:**
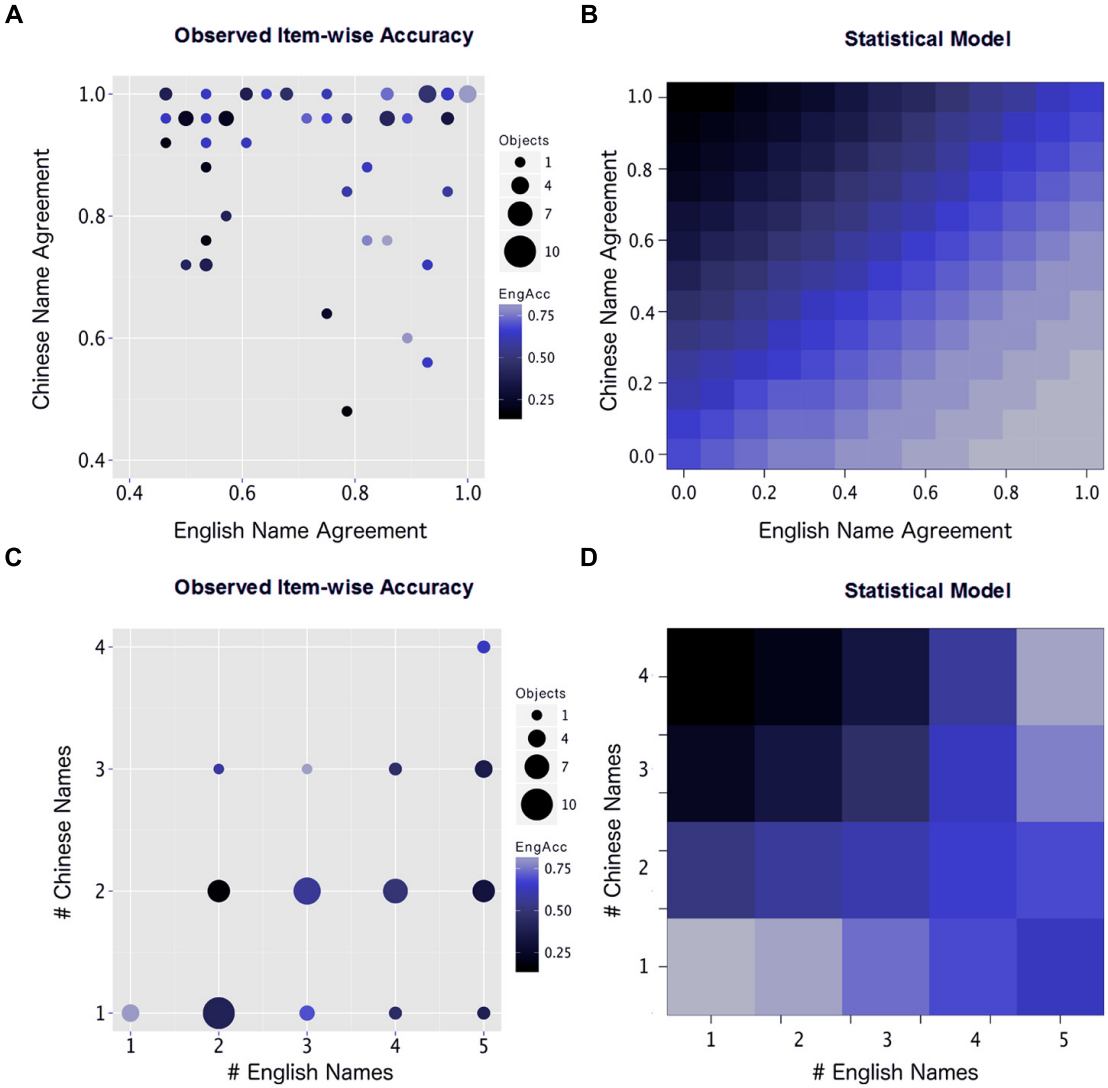
**Observed and estimated effects of language-specific variables: dominant name agreement and number of alternate names. (A)** The interaction between native speaker naming agreement in Chinese and English as predictors of English naming accuracy by Chinese–English bilinguals. Each point on this graph represents a set of objects with particular agreement values in Chinese and English. Color of each point indicates mean accuracy ratings for the participants, and size of each point indicates the number of objects represented. **(B)** The accuracy values estimated by the statistical model at varying levels of Chinese and English agreement, controlling for all learner variables and number of alternate names. **(C)** The interaction between the number of names generated by native speakers of Chinese and English as predictors of English naming accuracy by the Chinese–English bilinguals. **(D)** The accuracy values estimated by the statistical model at varying numbers of alternative names in Chinese and English, controlling for all learner variables and the level of native speaker agreement.

**Figure 5** depicts both the observed item-wise accuracies (A and C) and the estimated marginal mean accuracy at varying levels of the predictors using the logistic regression model. In **Figure 5A**, the average response accuracy across participants is plotted over both English and Chinese norm agreement levels for each object in the stimulus set. This plot shows the empirical effects observed in our sample and is generally consistent with the competitive account of L1 and L2 agreement estimated by the regression model. Among the objects in the experimental stimulus set, best performance (depicted by blue-colored dots) is observed when *both* languages have high agreement levels. Items in this set are not limited to one-to-one translation pairs, as *cup*, *mug*, and *glass* were all represented in this set (and all translated as *bēizi*).

This performance diminishes as English agreement decreases. In general, high levels of L2 (English) agreement are associated with successful learning across varying levels of L1 (Chinese) agreement. However, the worst performance by the learners occurs when Chinese agreement is high and English agreement is low, confirming that L1 patterns can have a strong negative effect on L2 native-likeness when L2 input is inconsistent. Many of these items came from the *bēizi* (roughly, *cup*) category, highlighting learners’ weakness with the sub-divided English categories it includes (*cup, mug*, and *glass*), but several cross-cutting categories that did not have clear one-to-one translations also appeared: *gāng*, *pénzi*, and *wăn* are approximately translated as decreasing sizes of *bowls* but are translated as *dish* for some items according to the monolingual norms.

For comparison, **Figure 5B** plots the model’s estimated accuracy levels, generalized to all levels of agreement in each language. While **Figure 5B** covers a broader range of potential values (such as low naming agreement in Chinese, which is under-represented in the actual stimulus set), it generally fits the patterns established by the empirical data. Further, the observed data (**Figure 5A**) do not control for confounding variables (such as the number of alternate names). The regression model estimates both predictors and thus isolates the effect of agreement while holding the number of names constant, resulting in the smoother contours along values of L1 and L2 agreement depicted in **Figure 5B**.

Participants’ observed performance across the different numbers of alternative names in the English and Chinese norms, however, differed significantly from the regression model’s estimated accuracy rates. **Figure 5C** depicts the accuracy rate for each object in the stimulus set across varying numbers of alternate names in each language. This plot indicates that when learners had only one L1 name for an object in each language, performance was highest. The worst performance was observed when exactly two competing names for an object were available in either language. The model’s prediction that more competing L2 names improve the probability of learners producing the L2 dominant name (**Figure 5D**) is consistent with the latter observation that objects with three or more competing names were named more accurately than those with two names, but it overlooks the advantage for objects with only one name. Again, in the observed data (**Figure 5C**), the number of names and agreement level are confounded, but the regression model isolates these effects and controls for agreement in its estimations of the effects of L1 and L2 names (**Figure 5D**). It is not clear that these representations disagree, *per se*. Rather, **Figure 5C** represents the objects provided in the stimulus set, while **Figure 5D** provides a controlled, parametric representation over many values of each variable. The discrepancy between these representations is further discussed below.

## DISCUSSION

### SUMMARY

In this study we examined the relative effects of four language history variables in predicting learners’ outcomes in L2 lexical categorization native-likeness. Highly significant interactions were found among these variables, supporting the idea that language history (e.g., age of L2 onset) variables should not be evaluated in isolation from other variables. Significant age of L2 onset effects were observed, but these effects were tempered by the positive contribution of increased immersion experience. A surprising observation was that increased experience with L2 prior to immersion was actually associated with reduced native-likeness of L2 lexical categorization. Finally, we found that for bilinguals with long-term L2 immersion, patterns of language use (i.e., code-switching habits) were a significant predictor of L2 native-likeness, but for learners with less immersion experience (including no immersion experience), language use was a less important predictor of L2 native-likeness.

We further explored how the naming norms of the linguistic communities of both languages influenced the learners’ success in acquiring native-like L2 lexical semantic mappings. Both L1 (Chinese) and L2 (English) norms were significant predictors of the learners’ L2 native-likeness, consistent with previous findings in other domains of second language acquisition, such as phonology. Further, we identified unique effects for agreement among native speakers and the number of alternate names produced in the norming samples. The result of an item-wise analysis revealed that a large amount of the between-object variation in naming was captured by these native speaker naming norms, indicating both the lasting impact of L1 mappings on L2 production and the sensitivity of L2 learners to the native speaker norms of the L2. Below we present a more detailed discussion of how L2 naming patterns are influenced by the learner variables and input (linguistic community norm) variables.

### LEARNER VARIABLES

#### L2 training

The most surprising finding of this study was that the number of years spent studying English outside an immersion environment was negatively related to L2 native-likeness in the lexical categorization task, even after controlling for the length of eventual L2 immersion. This outcome was not predicted by any past research nor intuition. This novel contrast between years of non-immersed and immersed learning in learners who have significant experience in both environments suggests that L2 training outside of an immersion environment may ultimately reinforce lexical semantic mappings that significantly differ from those of L2 native speakers. There is little doubt that immersion experience is beneficial to second language learning, and second language acquisition research has long promoted this view, but the present study adds the unique corollary that L2 learning without immersion may, in fact, hinder native-likeness. This effect may be due to the entrenchment of L1 structures in learners’ L2 as a result of impoverished input. Common classroom techniques for learning translation equivalents or naming highly prototypical objects encourage learners to export their inferences about object categories from L1 to L2 by way of one-to-one translation. However, native-like L2 mappings only become available to the learner with more diverse input from an immersion environment or (potentially) another immersive instructional setting such as the highly enriched virtual environments that may be simulated in computer games (see Legault et al., 2014, for example). The more time that L2 learners spend learning lexical semantic mappings in a non-immersive environment, the more entrenched the L1-driven mappings in L2 may become. Considered against this perspective and the relative proportion of L1 vs. L2 input in the non-immersive environment, the patterns in our data become less surprising but provide an important lesson for language instruction practice.

#### Age of onset

Second language lexical learning has often been regarded as a qualitatively different type of acquisition from phonology and syntax that tend to show strong age effects. One theoretical account, Ullman’s (2001) Declarative-Procedural (D-P) Model, attributes this dissociation to differences in the underlying memory systems that support lexical learning and all other aspects of language. This theory is consistent with observations to date about both native and second language acquisition, but the present findings suggest that the dissociation may not be so clear cut. When we measure lexical semantics as a complex system of mappings for making generalizations rather than just a set of word-object pairs, as in the present study, a weak pattern of age effects is replicated.

Age of second language onset effects may also be confounded with the negative pre-immersion learning effect. In the present study, we surveyed participants’ earliest exposure to English as a second language rather than their earliest immersion experience in an English language community. Although there were significant advantages for earlier learners over later learners, these advantages were limited in the sense that for every year of earlier acquisition, the same effects could be gained by an additional year of L2 immersion. With a small age of onset advantage on the one hand, and a non-immersed L2 learning disadvantage on the other hand, one may ask whether earlier L2 instruction is indeed beneficial for lexical semantic native-likeness. Addressing this question requires considering the multiple influence of both age effects, amount of total training, and the eventual onset of immersion (if at all). In a later section on implications for L2 instruction, we address these issues in further detail.

#### Code-switching frequency

Whereas age effects and training effects focus specifically on the conditions under which learners begin acquiring a new language, eventual native-likeness may just as well depend on how that language is used at later stages, such as in an L2 immersion environment. Switching from one language to another may be common, even difficult to avoid, in bilingual environments, but considerably more variation in individual CSFreq could be observed among bilinguals in relatively monolingual-like environments. While some bilinguals may use each language in a distinct context (e.g., home vs. work), others may switch frequently. Research in first language lexical attrition has highlighted the role of bilinguals’ specific language use patterns in re-shaping L1 (De Leeuw et al., 2010) and offered a cognitive explanation for how L2 structures are eventually encoded into L1 representations (Wolff and Ventura, 2009).

In the present study we observe a complementary effect. Increased code-switching is associated with greater L2 native-likeness. However, this effect interacts with L2 immersion such that it applies only after a significant period of immersion (illustrated at 4.7 years in **Figure 2**). For learners with significantly less immersion (including no immersion at all) code-switching behavior had no strong effect on L2 native-likeness, both emphasizing the importance of prolonged L2 exposure for the acquisition of these lexical semantic mappings and perhaps mitigating a belief that frequent switching between languages significantly impedes native-like acquisition of an L2.

The causal relationship between CSFreq and native-likeness cannot be determined from our results, however. One explanation would argue that increasing an advanced learner’s code-switching leads to improvements in L2 native-likeness by promoting simultaneous activation and therefore increasing opportunities for lexical semantic remapping. On the other hand, bilinguals with greater L2 native-likeness may already be more involved in bilingual social settings (as opposed to seeking out L1 contexts) and increase their rate of code-switching as a result. Future research could investigate the short-term effects of code-switching in an experimental procedure, but the long-term causal relationship between these variables remains unknown.

### LANGUAGE VARIABLES

Although the learner-oriented variables as discussed above proved useful in predicting overall performance in lexical categorization, they were rather inadequate in predicting native-like naming for individual objects. Language-specific variation, on the other hand, proved extremely important in predicting trial-by-trial accuracy of participants’ object naming, even after controlling for inter-participant differences in the learner variables. These effects have been revealed by our item-wise analyses. One lesson from these effects is that any kind of overall attainment score in lexical categorization masks significant variation in mastery for individual words, with some words posing much greater challenges for the learner (see also Malt and Sloman, 2003). The current data help reveal the source of the variation.

#### Native speaker agreement

We found a competing relationship between the level of native-speaker agreement in L1 and L2 in predicting the native-likeness of learners’ L2 responses. The role of L2 agreement in learners’ responses indicates that these learners are sensitive to variation in native speakers’ lexical categories for these objects. In the alternative case, where learners rely only on a general majority name for objects, we should see little effect of the L2 agreement variable, as learners would be more consistent than native speakers. Instead, learners respond proportionally to native speakers in their level of naming agreement. Further, the interaction between immersion and L2 agreement demonstrates that the advantage for high L2 agreement increased with greater immersion: These objects show greater improvement than low L2 agreement objects, which did not improve much even with almost 5 years of immersion.

Conversely, agreement among native-speakers of the L1 significantly impeded native-like naming in the L2, indicating that L1 learners were more resistant to revising their lexical semantic mappings in L2 when L1 native speakers were more consistent, likely showing a higher degree of confidence about the object’s category membership. The interaction between immersion and L1 agreement demonstrates that this L1 disadvantage predicts learners’ improvement with greater immersion experience. Low L1 agreement words significantly increase in their native-likeness with longer periods of immersion, while higher L1 agreement words show less improvement, highlighting these lexical semantic mappings’ resistance to restructuring.

Re-examining the observed accuracy rates for the learners across both L1 and L2 agreement levels, we found an antagonistic interaction between these variables. When L1 agreement was especially high, learners struggled to produce native-like L2 names, even at relatively high levels of L2 agreement. However, when L1 agreement was relatively low, L2 agreement was a better predictor of L2 learners’ native-likeness, apparently becoming more salient in the absence of strong L1 cues. The statistical model did not find such a strong interaction, instead identifying the same opposing main effects of L1 and L2 agreement but without an effect of the very small (though significant) interaction term. It remains to be seen whether the observed interaction is a byproduct of the objects in our particular task or whether the model simply underestimates the importance of this interaction. In either case, the important roles of L1 and L2 agreement norms are apparent, either independently or interactively.

#### Alternate names

The number of alternate names for an object produced by native speakers in L1 and L2 were also significant predictors of L2 learners’ native-likeness and were highly interactive with one another. If learners have only one name for an object in their native language, the model indicated that they would be equally likely to produce the dominant L2 name, regardless of alternatives. However, the observed naming behavior indicated that this trend overlooked a significant variation from L2 norms in the learners’ naming. For this subset of objects with only one name in L1, the lowest probability of producing a native-like L2 name occurred when the L2 provided two name alternatives, with greater L2 native-likeness occurring when only one L2 name was available or when three or more L2 names were available. This pattern suggests two mechanisms: (1) the attraction of the 1-to-1 translation, as learners struggle with competing pairs of L2 names, and (2) the competition within a distribution of L2 names, as learners’ performance improves with a greater number of name alternatives, showing some indifference to the L2 alternatives when there are several.

In the remaining conditions, when learners have multiple L1 words for an object, a greater number of L2 names appears to offer an advantage in selecting the dominant name. One potential explanation for this effect is the proportion of input that each alternative name comprises for L2 learners. Because the present model looks at both agreement in the dominant name and the number of alternatives, the latter provides an indirect measure of the native-speaker agreement levels for each alternate (non-dominant) name. As the number of alternative names increases, the remaining portion of the norm is divided into smaller parts relative to the dominant name, and thus each alternative name becomes a less salient competitor.

Under the foregoing explanation, we would expect the lowest L2 learner performance to occur when naming agreement is low *and* split with only one alternate name which shares all of the remaining native speaker agreement, e.g., 60% of native speakers name an object *plate* while 40% call the object *dish*. As new name alternatives are introduced, the second- through nth-most dominant names fall off in agreement, e.g., 60% of native speakers call an object *plate*, while 20% call the object *dish*, and 20% call it *platter*. This account of agreement and alternate names emphasizes the competition between names, and successful native-like naming is supported by greater agreement in the dominant name *relative* to the alternate names available. In our sample of objects, naming agreement and number of alternate names are correlated such that higher agreement is associated with fewer alternate names, producing the low performance effect at two alternate names (**Figures 5A,C**). The logistic model, on the other hand, dissociates these two variables and thus does not show this effect in either variable independently (**Figures 5B,D**).

As the number of names in the sample of L1 native speakers increased, L2 learners’ native-likeness declined, suggesting that the relative frequency of the dominant name was less important for L1 than the full array of available names. This observation makes intuitive sense, as we would expect the learners to have a more stable, entrenched knowledge of their native language. In the case of L1, participants may simply be sensitive to the presence of names regardless of agreement level, or alternate names in L1 may reflect a more general uncertainty about the identity of an object and, apart from language, its membership in semantic categories with other objects. If the function of an entirely novel object is unknown, even native speakers will have a difficult time settling on the best name for that object because lexical categorization does not strictly adhere to similarity of physical features like size and shape.

Finally, performance on six objects that had two competing names in both L1 and L2 was observed to be the worst overall among the stimuli. This effect is not replicated in the modeled plots because, again, it depends not only on the number of names but on the combination of name agreement and number of names, while the model parametrically varies each of these factors. Indeed, the item-wise observations are consistent with the proposal that the distribution of naming agreement between the two objects in L2 drives the general disadvantage for two-named objects.

### RELATIONSHIP TO PREVIOUS MODELS

In the introduction, we explored how theories of lexical and semantic representation could be extended to understanding patterns of lexical categorization. The present study does not directly implement any specific theoretical model, as we observe only the naturally occurring shifts in lexical categorization by Chinese–English bilinguals over their varying language learning and language immersion experiences. Nonetheless, connectionist theories such as the Distributed Feature Model (Van Hell and De Groot, 1998) and computational models (e.g., McClelland and Rogers, 2003) present a useful formalization for how word-feature mappings may be represented and adjusted with simple associative training paradigms (e.g., pairing the word *bottle* with feature sets describing its exemplars).

Specifically, important factors in connectionist training paradigms, such as amount, frequency, and consistency of input are readily translated into the lexical categorization terms used in this study. We quantify the amount of L2 experience (LOR), frequency of the dominant name relative to other names (naming agreement), and alternate names, finding compelling parallels between the associative learning principles that underlie connectionist models and the estimated effects of these variables on L2 categorization. For example, the (weak) age of onset effect observed in the present study concurs with entrenchment accounts of age effects in models of lexical acquisition (e.g., Li et al., 2007; Zhao and Li, 2010).

Entrenchment also provides some explanation for the relative disadvantage in re-mapping L2 categories for objects with high L1 agreement, as high agreement confers greater training frequency for the dominant L1 name (for a given object presentation). The role of L2 linguistic community variables in predicting learners’ native-likeness confirms that learners are sensitive to the relative frequency of several alternate names, showing improved performance when the agreement for the dominant name increases and decreased performance when alternate name competitors increase in frequency (e.g., two names distributed 60–40% versus three names distributed 60–20–20%). We also found support for the interactive relationship between L1 and L2 mappings, as suggested by the models proposed by Van Hell and De Groot (1998) and Dong et al. (2005). Future research may test whether or not manipulating these training parameters produces analogous results in computational simulations of category learning, validating the comparison between lexical categorization in language and lexical semantic learning in connectionist models.

### IMPLICATIONS FOR SECOND LANGUAGE INSTRUCTION

The present study offers several new insights into the role of language history, language training, and language use in second language lexical semantic learning. Most importantly, we find that greater time spent studying a second language before immersion predicted lower levels of eventual L2 native-likeness, likely due to the entrenchment of L1-like lexical-semantic mappings. Although we do find an age of onset effect, even after controlling for immersion and duration of language training, the magnitude of this age effect is proportional to the benefits of immersion, and the benefits to L2 native-likeness from early age of onset are small relative to the effects of more pre-immersion training.

On the extreme end, one might propose that pre-immersion language instruction is actually counter-productive to native-like lexical semantic development, and second language education would be best postponed until immersion opportunities arise. However, this viewpoint is impractical for most non-immigrant learners, and likely over-stated, as our analysis of language-specific variables (native-speaker agreement and alternative names) show that learners are, in fact, highly sensitive to the inconsistent input that describes native-like lexical categorization. Lexical semantic learning in non-immersion environments might therefore be improved by introducing learners to a greater variety of referents and the naturally diverse naming patterns associated with those referents, allowing them to develop more native-like intuitions about the relationships between objects that define lexical categories. The method of using a diverse set of naming patterns in second language instruction clearly contradicts the traditional classroom teaching method, in which training focuses primarily on one-to-one translations; such a focus underestimates cross-languages differences, and by our findings, encourages the use L1 patterns for L2 words and therefore impedes learners’ later ability to acquire native-like lexical semantic mappings.

## Conflict of Interest Statement

The authors declare that the research was conducted in the absence of any commercial or financial relationships that could be construed as a potential conflict of interest.

## Footnotes

1 Two participants in the US and four in China were excluded for response rates below 50% on one or more of the naming tasks. Non-response rates were approximately the same between the English task (four participants in Beijing) and the Chinese task (three participants in Beijing and two in State College), suggesting that most of these missing data were attributable to participant inattention. Two participants in China were removed for naming accuracy scores more than 2.5 standard deviations below the mean (see Subject-wise Analysis).

2 In two cases, the naming agreement score for an object was tied between two names. For each case, we randomly selected one name as the “dominant” name for the purpose of the comparison. This uncertainty, however, is preserved in the L2 Name Agreement variable included in the logistic regression.
